# Decoherence Principles and Algorithms for One-Dimensional Nonuniform Sampling Schedules for Multidimensional NMR

**DOI:** 10.1021/acs.analchem.5c03754

**Published:** 2025-12-05

**Authors:** Henry B. Rovnyak, Lucille E. Cullen, David Rovnyak

**Affiliations:** † Department of Chemistry, 4517 Bucknell University, Lewisburg, Pennsylvania 17837, United States; ‡ Purdue University, West Lafayette, Indiana 47906, United States; § Piedmont Virginia Community College, Charlottesville, Virginia 22902, United States

## Abstract

Nonuniform sampling (NUS) NMR is a potent method for enabling diverse multidimensional NMR spectroscopies, but it depends on the quality of the sampling schedule, particularly for one-dimensional NUS (i.e., for 2D-NMR) and for sparser sampling where noise-like artifacts (aka sampling noise) are commonly observed. Current NUS scheduling algorithms, while generally effective, can also allow flaws that lead to increased artifactual noise in spectral reconstructions. Computation or expert user curation can improve such schedules but are not easily reproduced at the spectrometer. This work builds on lessons that reducing patterns in NUS schedules can reduce artifacts and aid sparser NUS. It is proposed here that patterns in a sampling schedule be treated sequentially at local and global scales. First, a localized decoherence filter is presented that leverages the properties of the binary Thue–Morse (TM) sequence to remediate patterned subsequences in the schedule. Next, an approach to polishing the point-spread-function (PSF) by an iterative thresholding method was developed, where improving the PSF treats the schedule globally. These algorithms are implemented in a hands-free scheduler for one-dimensional NUS and tested with both iterative soft thresholding (IST) and iterative line shape (SMILE) reconstructions. While varying degrees of sampling noise are still expected, particularly in sparser NUS conditions, these methods reduce larger spectral artifacts and perform more consistent design of schedules for broader use, as illustrated with sodium naproxen, strychnine, and u-^13^C,^15^N-ubiquitin for weighted (e.g., quantile, Poisson gap, exponential), and random unweighted (RU) NUS, though limitations of sparse RU-NUS should be considered.

## Graphical abstract

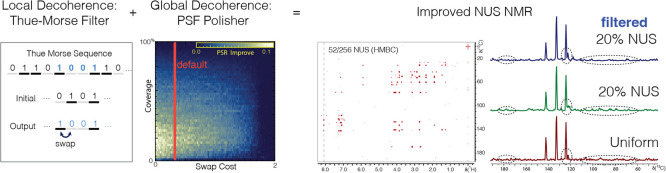

## Introduction

Nonuniform sampling (NUS) reduces the number of incremented steps needed for multidimensional NMR and is used for substantial time and cost savings, resolution improvements, and some applications to moderately improve sensitivity.^1^ While its use has become relatively routine in high dimensionality biomolecular NMR, NUS for diverse time-consuming 2D-NMR experiments is often restricted to conservative regimes (e.g., 50% reduction). Acquired NUS data are specified by a sampling schedule, where a distinction has arisen between one-dimensional NUS (1D-NUS) schedules for 2D-NMR and higher dimensional NUS schedules (e.g., 2D-NUS for 3D-NMR). In the latter case, signals are distributed over additional dimensions that help to meet important sparsity conditions for spectral reconstructions;^2^ further, nD-NUS schedules (*n* ≥ 2) can be robust to flaws in a given schedule row or column likely due to nearby samples, although the role of neighbor samples in nD-NUS schedules requires more study. In contrast, using 1D-NUS in 2D-NMR can face high signal densities and can be unforgiving to flaws in the sampling schedule.

Reducing the number of samples in the indirect dimension/s of nD-NMR imposes two constraints: the characteristics of the reduced set of samples to be acquired (i.e., of the sampling schedule), and the fidelity of the subsequent spectral reconstruction or parameter extraction methods. Given mature and emerging methods for spectral estimation,^1^ considerable work has been dedicated to the unique challenges in designing and improving 1D-NUS schedules,^3–16^ the focus of this work.

The use of 1D-NUS in 2D-NMR must consider sampling noise/artifacts^17^ and the potential for insufficient samples.^2,18^ Sampling noise can arise from Fourier components of the sampling schedule,^1,19–21^ and may be nearly fully removed in conservative NUS, but is more common in sparser NUS. Further, NUS does not enjoy the full protection of the Nyquist theorem, where artifactual noise can also arise from aliasing of signals inside the spectral window.^8,22–24^ Any sampling schedule has a likelihood to contain patterned subsequences (coherent sampling in time) that can lead to unwanted artifacts in subsequent reconstructions, where reducing such patterns (decoherent sampling time, i.e., decoherence) can improve spectral quality and help achieve sparse or even ultrasparse NUS^8^ (Supporting Information).

Methods for generating 1D-NUS schedules include exponentially weighted sampling,^10,17,25^ quantile sampling (QS),^6^ Poisson gap sampling (PG),^5^ and schedule averaging,^26^ and were designed in part with the intent to reduce sampling noise and promote decoherence. Users should be aware of the separability of sampling concepts: a choice of weighting function and a strategy for selecting the samples. A complicated default weighting function has been associated with the PG method.^12^ The QS method is offered with several choices of weighting functions,^6^ where this present work recommends a default sine-chord weighting. Anticipating notational recommendations at a later time, this report will use QS and PG to imply their default weighting options (see Methods for elaboration). While these methods can produce strong 1D-NUS schedules, analyses of the PG and QS methods found that their base algorithms can sometimes produce schedules that lead to greater spectral artifacts, where both computational^7^ and metric-driven^8^ methods were devised to improve such schedules. The former screened seeds to reduce artifacts;^7^ the latter enforced decoherence and screened the point-spread-function (PSF).^8^ Both efforts yielded fixed schedules for broader use. Recently, PG schedules were screened with *NUSscore* ^27^ for their utility in NOE spectroscopy.^28^

The curated two-step procedure of Cullen et al.,^8^ in which an initial schedule was first iteratively tested for patterned subsequences and then screened for outlying point spread function (PSF) features to examine it holistically, showed that reducing local and long-range order in 1D-NUS schedules is feasible and confers improvements in 1D-NUS of 2D-NMR. However, that approach is curated and requires some user specialization in NUS. We also caution that the connection between decoherence and spectral quality is nuanced, since patterns can occur in varying degrees and complexities, while short patterned regions can even confer useful properties.^8^

The present work pursued new theory and methods to objectively and hands-free generate decoherent 1D-NUS schedules for nD-NMR. We first propose a new decoherence filter that does not rely on randomness and exploits useful properties of the binary Thue–Morse (TM) sequence,^29^ termed a “Thue–Morse decoherence filter” (TM filter). This TM filter remediates local patterned regions of the schedule, but does not directly treat preexisting global patterns, and can even contribute to global biases in a schedule. Next, a method to treat global patterns was devised by improving the schedule’s PSF, since PSF features are determined by the whole schedule. Some metrics based on the PSF and its sidelobes are not helpful,^15,28^ highlighting a challenge to better utilize the PSF. To address this need, a new approach to smooth the PSF with soft thresholding methods was devised, termed here a “PSF iterative thresholding Polisher” (PSFP).

The complete two-step filtering procedure still honors the characteristics of the initial schedule and is denoted by superscripting the “TMPF” acronym (TMPF = TM + PSFP) to the name of the base algorithm. By example, the sequential application of the TM and the PSFP algorithms to initial quantile or Poisson gap schedules can be termed QS^TMPF^ and PG^TMPF^, respectively.

Sampling noise/artifacts in reconstructed NUS spectra result from reduced sampling, regardless of the detailed properties of the schedule. The decoherence methods presented here lead to more reliable schedule generation, artifact reduction, and accessing sparser regimes, but leave open questions. For example, to what degree should patterns be reduced when producing sampling schedules for NUS NMR?^8^ And how best should short initial uniform regions be used? ^8,13,22,23,28^ Considering the diversity of 2D-NMR experiments, the desirability of hands-free automation in the face of numerous NUS parameters, and favoring conservative choices, the use of very short initial uniform sequences was continued in this work, but begs additional inquiry. A complete hands-free scheduling program “*Usched*” is available (on NMRbox and gitlab) to generate 1D-NUS schedules by the methods presented here.

## Methods and Experimental

All NMR spectra were acquired on a 600 MHz spectrometer (Varian Inc., DDR1) with an inverse room temperature probe at 25 °C and using vendor-supplied pulse sequences without further modification. Typical pulse widths were p­(p/2, ^1^H) = 7 ms and p­(p/2, ^13^C) = 28 ms. Sodium naproxen and strychnine were purchased from Sigma and used without further purification. The u-^13^C,^15^N-ubiquitin sample (∼1 mM) was purchased from Cambridge Isotope Laboratories.

### Schedules and Notation

In order to avoid preempting separate terminology efforts, we clarify two notational choices here. (i) The term Poisson Gap (PG) is used in this work to indicate a specific case of passing a sinusoidal weighting parameter to the Poisson deviate generator, where we stress that the resulting distribution of samples is *not* sinusoidal, but is a complex function that is strongly weighted to early times, followed by a long nearly uniform portion.^12^ (ii) Similarly, this work also employs the flexible quantile algorithm which can implement several weightings, but will use the term QS to denote the default weighting choice, which is a chord of the sine function.

### Processing

Spectra were processed either with MNova (v15.0.0, MestReLab Research), where all NUS spectra were processed with the “dynamic” MIST algorithm, or by the SMILE algorithm in NMRpipe.^30,31^ In both this (e.g., Figure S1) and prior work,^8^ we notice a tendency for the initial default “static” implementation of MIST to reconstruct aliasing (and potentially other) artifacts prominently. The initial application of the static MIST operates on the raw time domain data, but since IST is sensitive to the phase of the signals, it is the experience of this work that the artifacts were best reduced by phasing the initial static reconstruction and then enabling dynamic MIST to ensure the reconstruction is based on correct phases. A related rubric was followed in SMILE processing, which expects in-phase signals, such that spectra should be phased and reprocessed. Default parameters were used with SMILE, other than increasing maxIter until it exceeded the actual iterations and specifying nSigma = 3, where artifactual noise generally increased with nSigma = 4.

### Software

This work has generated a program “*Usched*”, written in the rust language and available for download on gitlab,^32^ which supersedes the prior “*Qsched*” software package that supported only quantile scheduling.^6^ The *Qsched* program remains available for download to ensure traceability of applications that have used it. The new *Usched* program is available on NMRbox.^33^

Further discussion of the algorithms used in this work is given in Figure S9 of the Supporting Information. The new *Usched* program implements the TMPF method developed here as well as a larger suite of base scheduling algorithms, including the quantile and Poisson gap algorithms as well as exponential and random unweighted schedules. The PG algorithm has been modified to guarantee the last point of the Nyquist grid specified by the user. The schedule averaging approach of Palmer et al. is included and was used for generating exponential schedules in this work.^26^

## Results

Results are organized around developing new decoherence filters for improving nonuniform sampling schedules and then testing their efficacy.

Designing sampling schedules has become more sophisticated as the criteria become better understood, and as more demanding applications of NUS are sought. Some tactics include weighted NUS for improved sensitivity,^25,34^ decoherence (pattern avoidance),^35,36^ and tessellation/gap management strategies.^5,6,12,37,38^ The choice of weighting function,^3,26^ partial component sampling,^35,39,40^ improving reproducibility and low-variance,^3,41^ and managing point spread function (PSF) characteristics (see Love et al.^15^ and references therein) have also been pursued. Further, the number of samples should be sufficient for the number of signals expected in the data.^2,18^ Finally, a schedule for general use should be agnostic to prior knowledge, although with experience the further design of schedules such as for sensitivity can be considered.^42^ Schedules may be developed for use with specific reconstruction algorithms.^7^

An additional consideration is to empower users, such as presenting choices of methods and metrics to users, providing robust default parameters, presenting users with a legible and intuitive algorithm, facilitating reporting, and broadening platform accessibility.^10^ Algorithms such as the Poisson gap (PG) or quantile sampling (QS) approach, can satisfy some of the criteria above, but methods for further improvements are sought.

### Treating Local Decoherence: Devising a Thue–Morse Filter

Patterns are a natural consequence of randomness.^43^ Whether nonuniform sampling is weighted or unweighted, patterns will emerge regardless of the base algorithm. We have developed both coarse binomial and more precise probabilistic treatments of patterns in weighted and unweighted sampling, presented in the Supporting Information (Figure S10). Prior work identified the alternating (1 0) repeat occurring commonly in the early weighted portion of the schedule and influencing spectral reconstructions.^8^ Pattern types and distributions can be complex, but remediating the (1 0) repeat was a proxy for pattern reduction in that prior work.

The Thue–Morse (TM) sequence is an infinite sequence in the binary alphabet, {0, 1}.^29,44^ The sequence can start with either 0 or 1 and is extended by appending the logical *not* of the prior portion of the sequence (Figure 1). The TM sequence resists patterns, where one of its features is that it contains no triples, meaning that for all binary strings “X”, the TM sequence does not contain “X X X”. For example, it does not contain the string “1 0 1 0 1 0” because it is a repetition of the string “1 0” three times.

**Figure 1:**
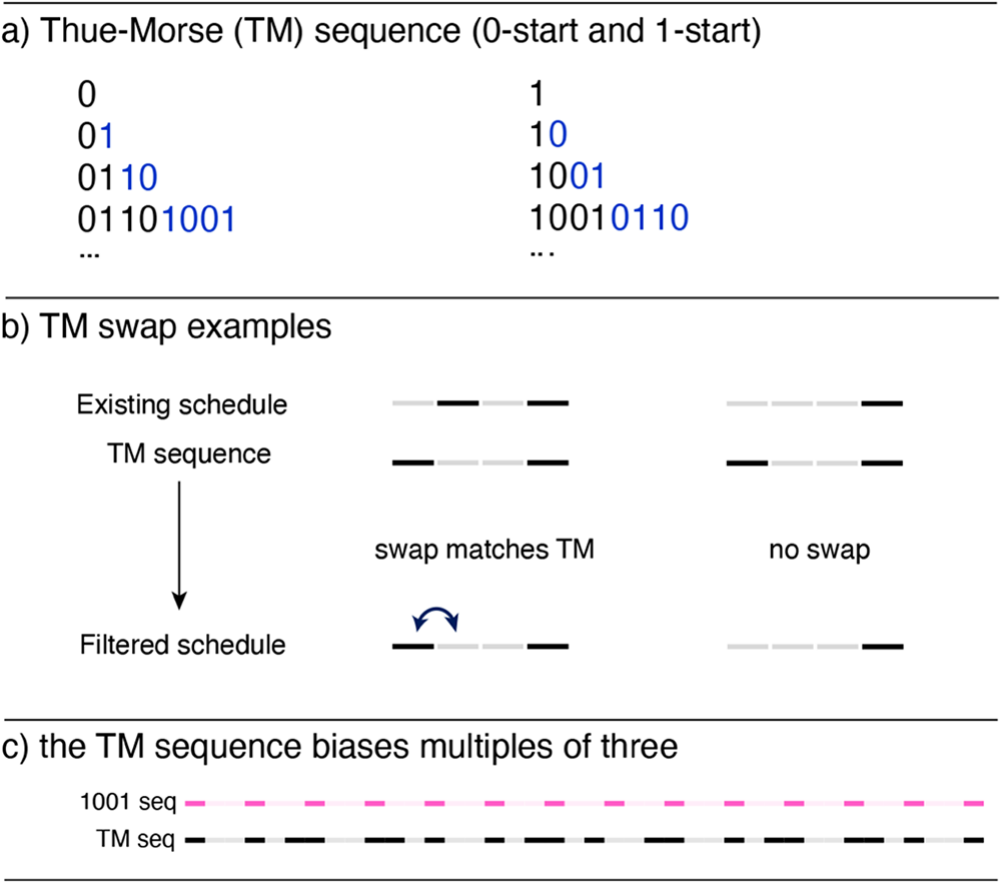
(a) The “0 start” and “1 start” Thue–Morse (TM) sequences; (b) the proposed concept of using TM sequences in a decoherence filter is shown, where gray and black bars represent 0 and 1, respectively; (c) an illustration of a region of the TM sequence (10010110..., black bars) that shows the bias toward multiples of three by comparing it to the 1001001... pattern (pink bars), highlighting that parts of the TM sequence overlap with the 1001001 pattern.

The TM sequence is not likely to be useful as a schedule directly since it is fixed at 50% coverage, has no weighting function, and can have poor PSF characteristics. It is proposed here to leverage the intrinsic decoherence of the TM sequence as a filter to amend an existing schedule. Ideally, a filter should match and change patterned regions of an existing schedule, without affecting the distribution of samples in the schedule. A swapping algorithm was devised in this work that accomplishes this goal of amending patterned regions, illustrated for two examples in Figure 1b. Specifically, we step the schedule through two-bit windows, comparing the schedule to an arbitrary slice of the TM sequence. If two consecutive bits of the schedule are the logical opposite of the two bits of the TM sequence, we swap the bits in the schedule to match the TM sequence at that index. Notice in the examples in Figure 1b that a short subsequence containing the {1 0} pattern is amended by the TM swapping algorithm, but a subsequence that does not have the {1 0} pattern is not altered.

The proposed algorithm performs two-bit swaps, so it can at most move a sample by one position, minimally affecting the intended sample distribution. A point cannot be swapped twice since, if it was swapped in one step, then the point would have been corrected to match the TM sequence and would not be swapped in the next step. The two-bit swapping algorithm honors the characteristics of the initial schedule, but foreshadows a potential limitation that will be shown later, that the TM filter cannot make large changes if an initial schedule is deeply flawed.

The TM filter algorithm has one parameter, an initiation location in the Thue–Morse sequence. This parameter is arbitrary and analogous to a random seed. The end point is determined by the length of the schedule.

We found that the Thue–Morse sequence has an implicit bias toward indices that are multiples of three (Figure 1c) that can carry over to the TM filtered schedule (not shown). In the resulting point spread function (PSF), such a bias can manifest as spikes at frequencies with wavenumber 1/3. The final schedule may not inherit much or any of this “1001” character if the TM filter does not need to provide many corrections. We first considered if the TM filter could be modified to avoid this bias when it occurs by testing window sizes or randomized TM slices to smooth out the bias, however the results were not satisfactory. We then turned to devising a dedicated method to treat global biases by smoothing the PSF, described next.

### Treating Global Decoherence: Iterative Thresholding PSF Polisher

As noted, the TM filter step may introduce weak long-range (i.e., global) biases. Equally important is that the initial schedule may also contain global biases. The point spread function (PSF) is the Fourier transform of the sampling schedule, and the PSF contains noise-like features that can “leak” into spectral reconstructions.^19^ Since the PSF is computed from the entire schedule, it is a potentially useful representation for addressing long-range (global) biases, but it remains enigmatic. The complex features of the PSF can obscure flaws,^8^ while certain metrics such as the peak-to-sidelobe-ratio (PSR) have limited or poor utility in screening schedules.^15,28^ Further, the power of the PSF is a constant for a given coverage (e.g., 25% NUS), so it is the distribution of the features of the PSF that must be considered.

We sought to devise an alternative approach to utilizing the PSF to evaluate and improve schedules. In brief, the PSF Polisher (PSFP) developed here makes targeted swaps on a schedule to most effectively suppress PSF artifacts, and has the effect of redirecting strong spikes (high power) into regions of lower power in the PSF, and acts as a smoother of the PSF (Figure 2). It iteratively calculates the swap that will most effectively reduce the amplitude of a thresholded PSF. That swap is applied and the updated schedule becomes the new input. The algorithm tracks which points have been swapped in order to not swap them again. The iteration will terminate when no more swaps can be performed. The initial schedule and the schedule generated after each iteration are tracked in a list and then scored to identify diminishing returns. The schedule with the lowest penalty score is returned.

**Figure 2:**
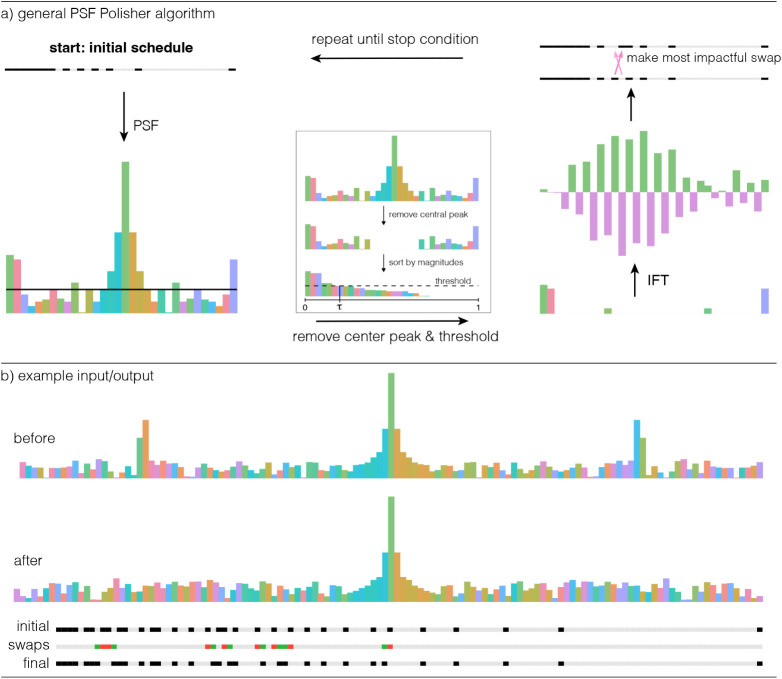
(a) The PSFP algorithm begins by calculating the PSF for the initial schedule. Next, the inset shows how the PSF is thresholded: the central peak is removed by stepping from the center outward until PSF features no longer decrease (i.e., stopping at the first nondecreasing value). Then, the remaining PSF signals are ranked, and a soft threshold is applied to identify the strongest features. The thresholded peaks are retained in the PSF, which is next subjected to the IFT to identify the region of the sampling schedule that relates to the thresholded PSF features; one swap is performed on two bits {1 0} that have the greatest difference in the IFT of the thresholded PSF, and the result then becomes the input for another pass until a stop condition is identified, which is illustrated separately in Figure 3. (b) The PSF polisher is seen to efficiently identify a small number of swaps that smooth even strong spikes from an initial PSF, while returning a schedule that closely resembles the initial input.

In the first stage of the PSFP (Figure 2), the PSF is calculated from the initial schedule, such as a quantile or PG schedule. Next, the central peak is removed and the remaining signals are ranked and subjected to a threshold to identify a subset of the strongest PSF peaks, which are not the central peak. The soft thresholding (inset, Figure 2) is determined by sorting the values of the PSF by decreasing amplitude, and setting the threshold to be the amplitude of the peak at index *k*, where *k* = [*n*,τ], *n* is the length of the schedule, and *t* is the “selection threshold”, which is a parameter of the algorithm that is fixed in default settings. The central peak of the PSF is removed prior to thresholding since it convolves with the true signal and does not need to be treated.

To determine the optimal swap to make in the sampling schedule from the thresholded PSF, we next compute the inverse-Fourier transform (IFT) of the thresholded PSF, resulting in a time domain PSF (td-PSF) that can be informally thought of as the “sampling schedule of the largest PSF features” (Figure 2). The amplitudes of adjacent values in the td-PSF are examined, where the most impactful swap is the one with the greatest difference in the amplitude of the IFT of a bit that is “1” in the corresponding sequence and an adjacent bit that is “0” (Figure 2). Intuitively, it is the swap that best “goes against the grain” of the IFT.

In order to seek the smallest number of swaps that smooths the PSF, the coverage of the sampling schedule must be taken into account and an exit condition devised. These tasks were enabled by incorporating a penalty score of each intermediate schedule as the sum of the peak-to-sidelobe ratio (PSR, utilized here as the sidelobe to central peak ratio so that it can be cast as a minimum) and the number of swaps applied, termed a “swap cost” parameter, which effectively defines how much of a PSR decrease is needed to justify one more swap. Further details of the procedure, with graphical illustration, are given in Figure S7 of Supporting Information. Performing the fewest swaps is important to mitigate the chance that the PSFP algorithm could make large changes to the sampling distribution or reintroduce patterns removed by the TM filter.

Iteratively suppressing the thresholded largest features present in a given PSF means that multiple spikes may be removed, and that their power will be distributed to other portions of the PSF, leading to a smoothing effect by the PSFP method (Figure 2). The PSR in the context of the PSF polisher reports on significant PSF changes. In contrast, comparing the PSR of two randomly generated schedules is a coarse parameter that is not likely to be useful, as has been shown.^15,28^ Empirically, the broader PSFP philosophy is to largely preserve the decoherence from the prior TM step by requiring relatively few swaps to ameliorate strong features in the PSF. Finally, it is reminded that the order of steps, the local TM filter followed by the global PSF polisher, reflects that the TM filter can introduce global biases which, if present, should be treated by the PSF polisher. See Figures S3 and S4 of Supporting Information for the distributions of swaps that are performed by the RM and PSFP steps.

### Spectral Reconstructions of NUS Data Employing the TMPF Decoherence Filter

The Thue–Morse filter and the PSF polisher are designed to be applied in series to address local and global decoherence (see also Figure S8 of Supporting Information) where together they are denoted by the superscript “TMPF”. As a part of this work, a default setting for the quantile sampling (QS) algorithm was devised that was used in all QS cases (see Methods).

In the development phase of this work, the TMPF filter was determined to strictly decrease the repeat length histogram (not shown), which can be requested by the user in *Usched* and which correlates to changing aliasing noise,^8^ as well as the PSR, which is a poor metric for evaluating initial schedules^15,28^ but takes on a new and potent role in the PSF polisher developed here. Decoherence is already known to be beneficial to NUS reconstructions, so this work sought to test if the new theory-based and hands-free TMPF methods for decoherence were sufficient to result in observable changes in diverse spectra.

The successive application of the TM filter and PSF polisher to an initial quantile schedule for an HMBC spectrum of sodium naproxen is illustrated in Figure 3. The initial schedule has resulted in a spectrum showing weak aliasing artifacts (circled, Figure 3a). The TM filter on its own reduced these artifacts in the reconstruction, but some strong artifacts remain (Figure 3a,b). The subsequent PSFP then produced a schedule for which the weak aliasing artifacts are suppressed, and which compares well to a uniform spectrum. For such low sparsity (20%) and low number of samples (52/256), it is reasonable that some artifactual noise is still distributed in the spectrum in Figure 3c, but the elimination of larger aliasing artifacts is demonstrated. These trends are also reflected in more detail in the ^13^C cross-section indicated (arrows) in Figure 3, where other artifacts are also reduced. Analogous behavior was seen when examining a PG schedule with the TM filter and the PSF polisher individually as well as in combination, shown in the Supporting Information (Figure S1). Additional control tests, notably to previously validated schedules, are also given in the Supporting Information (Figure S2).

**Figure 3:**
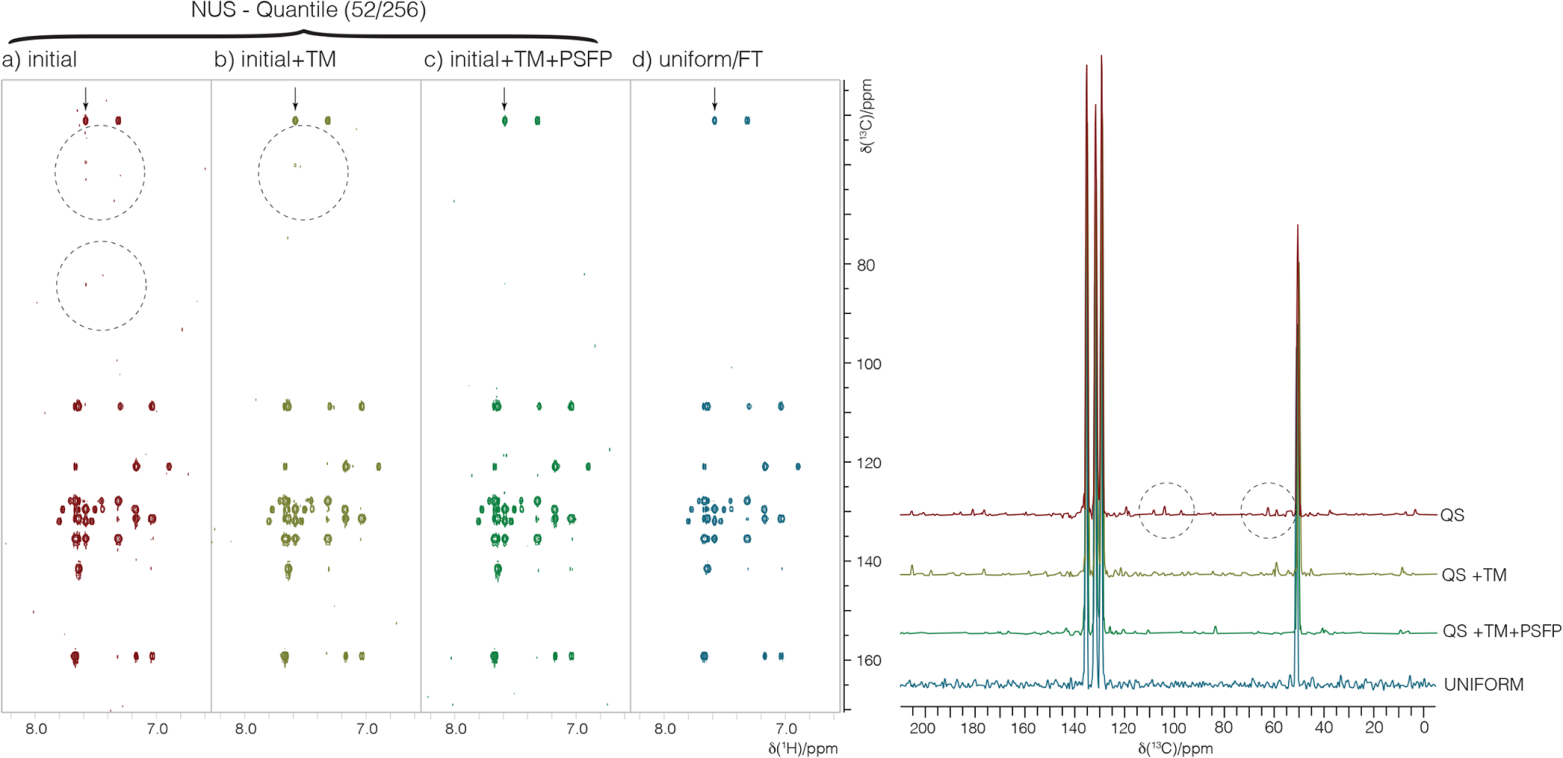
^1^H­(^13^C)-HMBC spectra of sodium naproxen (10 mM) obtained by IST reconstruction of NUS data and FT processing of uniform data. Spectra used 2 h of experimental time each. The sequential application of the TM and PSF polisher steps reduces larger artifacts in the reconstructions, particularly in regions prone to aliasing artifacts, such as at half the spectral window (dashed circled regions). A cross-section of the ^13^C dimension (see arrows on 2D plots) at the location of the circled artifacts shows in detail their progressive reduction with the TM and PSF polisher steps, where other noise spikes are seen to be reduced as well when using the TMPF schedule.

Conditions were sought that would incorporate sufficient artifactual noise into the initial spectral reconstructions to test the potential for artifact reduction after applying the TMPF method to the respective base schedules. When the number of nonuniform samples is limited compared to the maximum number of signals expected in the F1 slices, significant artifactual noise can be observed in spectral reconstructions, where a detailed study of NUS relative to signal density was reported by Nichols et al. for employing NUS in demanding 2D-NOESY and 3D-NOESY experiments.^18^ We selected 20% coverage (52/256) for strychnine HMBC spectra which has signal dense regions (up to about 6 signals per slice). Such conditions correspond to the approximate threshold for acceptable reconstructions determined by Nichols et al.^18^ Taking advantage of the ability to use random seeds with the Poisson gap method, eight independent trials with PG (52/256) were prepared with and without TMPF filtering, where three of the trials are shown in Figure 4.

**Figure 4:**
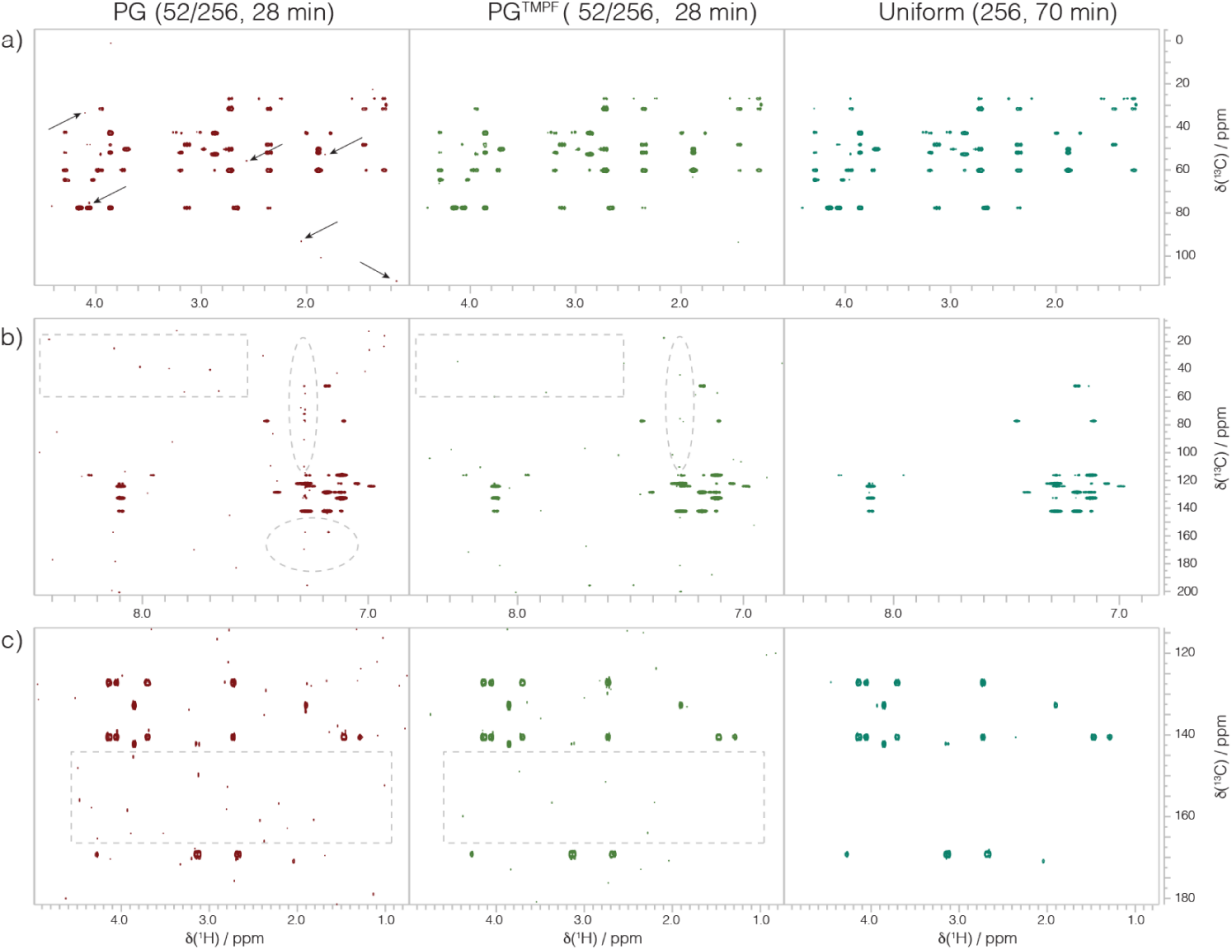
TMPF procedure was tested in conditions selected to produce artifacts (20% NUS, 52/256; see comments in text). Each spectrum used 28 min. Three examples of eight independent trials are shown in which base PG schedules with a random seed were generated and tested without and with the “TMPF” procedure developed here. Scans per transient were 4 for NUS and 8 for US. Spectra were processed to (4096 × 512) final size with cosine-squared apodization in each dimension; each row was subjected to automated normalization to facilitate comparison. Spectral windows were large enough to test for the appearance of aliasing noise, illustrated in (a). The TMPF algorithm reduced larger sampling artifacts in six of the eight trials and gave comparable sampling noise in the other two (not shown). The full uniform spectrum can be viewed in Figure S5 of the Supporting Information.

Three cases (trial numbers 2 (row a), 7 (row b), and 8 (row c)) are illustrated in Figure 4 that had more pronounced artifactual noise that were remediated by the TMPF filter. Trials 3, 5, and 6 (not shown) exhibited similar degrees of improvement to trials 2 and 8. In trials 1 and 4 (not shown), the PG^TMPF^ schedule resulted in fewer of the larger artifacts but did not appreciably change the broader background of artifacts. This series of PG tests (Figure 4) supports that the TMPF procedure improves schedules under challenging conditions and does not worsen schedules. It is stressed that the conditions used in Figure 4 will yield some degree of sampling artifacts in signal dense spectra regardless of the schedule, supporting Nichols et al.^18^ It is added that by amending the distinct initial PG schedules to perform similarly, the TMPF methodology promotes low-variance, namely that different schedules can yield similar spectral information.^3^

The generality of decoherence filtering should be tested more broadly, where we examined also the widely used SMILE (sparse multidimensional iterative line shape-enhanced) algorithm,^31^ implemented in the NMRpipe software suite.^30^ As before, challenging accelerated experiments (15 min, 25% coverage) were chosen that would be likely to produce artifactual noise for PG, QS, and random unweighted (RU) schedules in order to test the efficacy of the TMPF treatment (Figure 5). Interestingly, the QS tests were unremarkable and had generally lower artifacts in both the base and TMPF cases and are not shown. In other words, if the base schedule is already strong, the TMPF filter is not likely to lead to improvements. Differences between the base and TMPF schedules were consistently observed in the PG and RU tests, where insets confirm a theme that TMPF modified schedules suppress stronger artifacts. It is also seen that TMPF treatment tends to remodel noise, which we infer owes to smoothing PSF noise (Figure 5).

**Figure 5:**
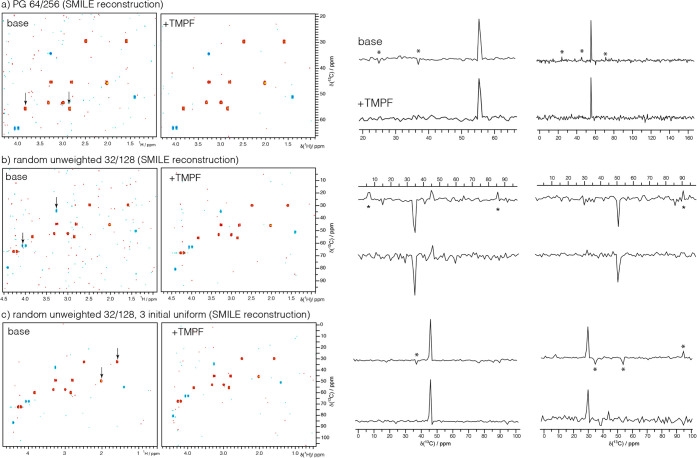
Smile reconstructions of base and TMPF-treated PG and RU schedules for ^1^H­(^13^C)-HSQC spectra, each requiring 15 min, of strychnine (10 mM) illustrate that larger artifacts in SMILE spectra using the base schedules tend to be reduced in TMPF-treated cases, where noise can be more distributed as well. Contour plots are normalized. Asterisks (*) denote larger artifacts.

To model the breadth of the TMPF scheduling algorithm, a test of QS^TMPF^, PG^TMPF^, and RU^TMPF^ schedules generated hands-free and used without any curation is shown for 20% HSQC spectra in Figure 6. In contrast to signal dense spectra in Figure 4, HSQC spectra will exhibit low counts of authentic signals in F1 slices (on the order of just 2 or 3 for strychnine at 600 MHz), so that the spectra in Figure 6 are a more conservative application of NUS.^18^ The spectra in Figure 6 are difficult to discern from each other, supporting prior work that coverage (sparsity) is a strong determinant of schedule performance, having more influence over spectral parameters than the specific base algorithm.^3^ The spectra in Figure 6 were peak picked to analyze differences, however peak positions (in ppm units) were virtually identical between the cases to at least four decimal places and no further comparison was deemed necessary. The inset shows two close carbon shifts that are well resolved in all cases.

**Figure 6:**
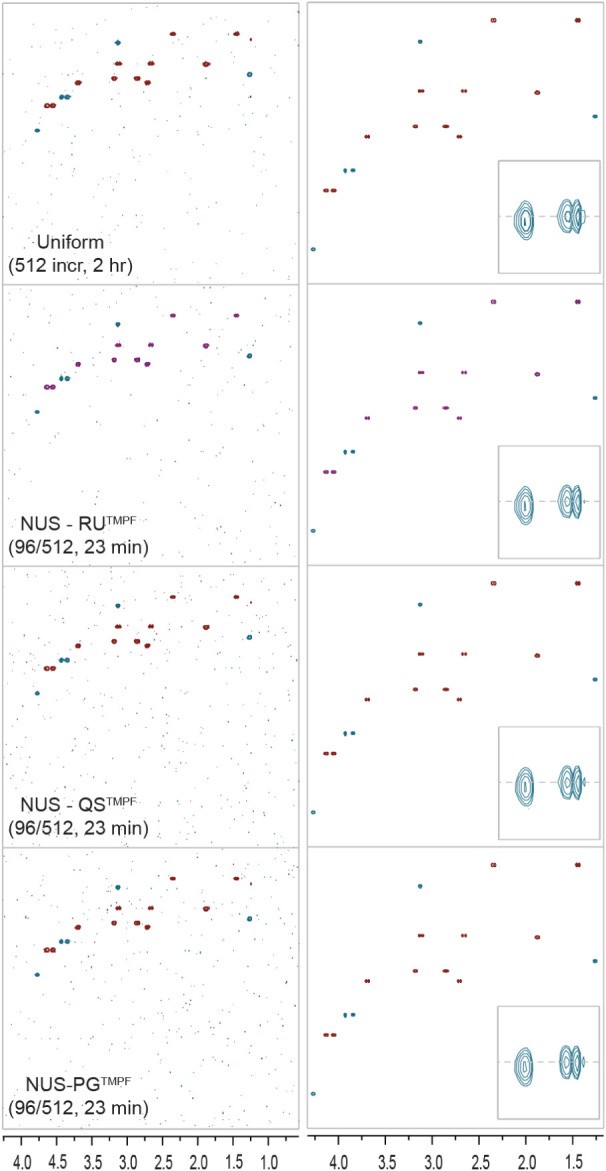
Hands-free generated schedules using the TMPF processing methods developed in this work are demonstrated on multiplicity-edited NUS–^1^H­(^13^C)-HSQC spectra of strychnine (9 mM) at about 20% coverage for time savings while spanning a long evolution period (MIST processing). A large F1 window demonstrates again that aliasing artifacts are not observable in F1, while progressively zoomed regions show good spectral quality. The final zoomed region in the inset of the right column shows preservation of spectral resolution in all approaches. The left column uses low contours to show the absence of aliasing artifacts in F1 and demonstrate the overall characteristics of the noise.

In this work, both QS^TMPF^ and PG^TMPF^ schedules obtained in the default hands-free mode of the algorithm performed well under a variety of conditions. In contrast, more complex and mixed results were obtained with random unweighted schedules, as illustrated in Figure 7 for an example employing ^1^H­(^15^N) HSQC of *u*-^13^C^15^N-ubiquitin. Recalling the good performance of the RU^TMPF^(128/512) schedule used above in Figure 6, a more challenging RU^TMPF^(32/128) schedule instead developed strong artifacts, including a small number that are comparable to authentic peaks in the resulting spectrum (Figure 7). Investigating further, we reached several empirical conclusions about random unweighted sampling: (i) RU schedules are less reliable than weighted schedules when the absolute number of samples is small and with sparser schedules; (ii) initial base RU schedules can have such serious flaws that the TMPF filter does not amend them fully; and (iii) whereas short initial uniform regions are still supported for weighted NUS, their role in sparse RU schedules requires more investigation (Figure S6, Supporting Information).

**Figure 7:**
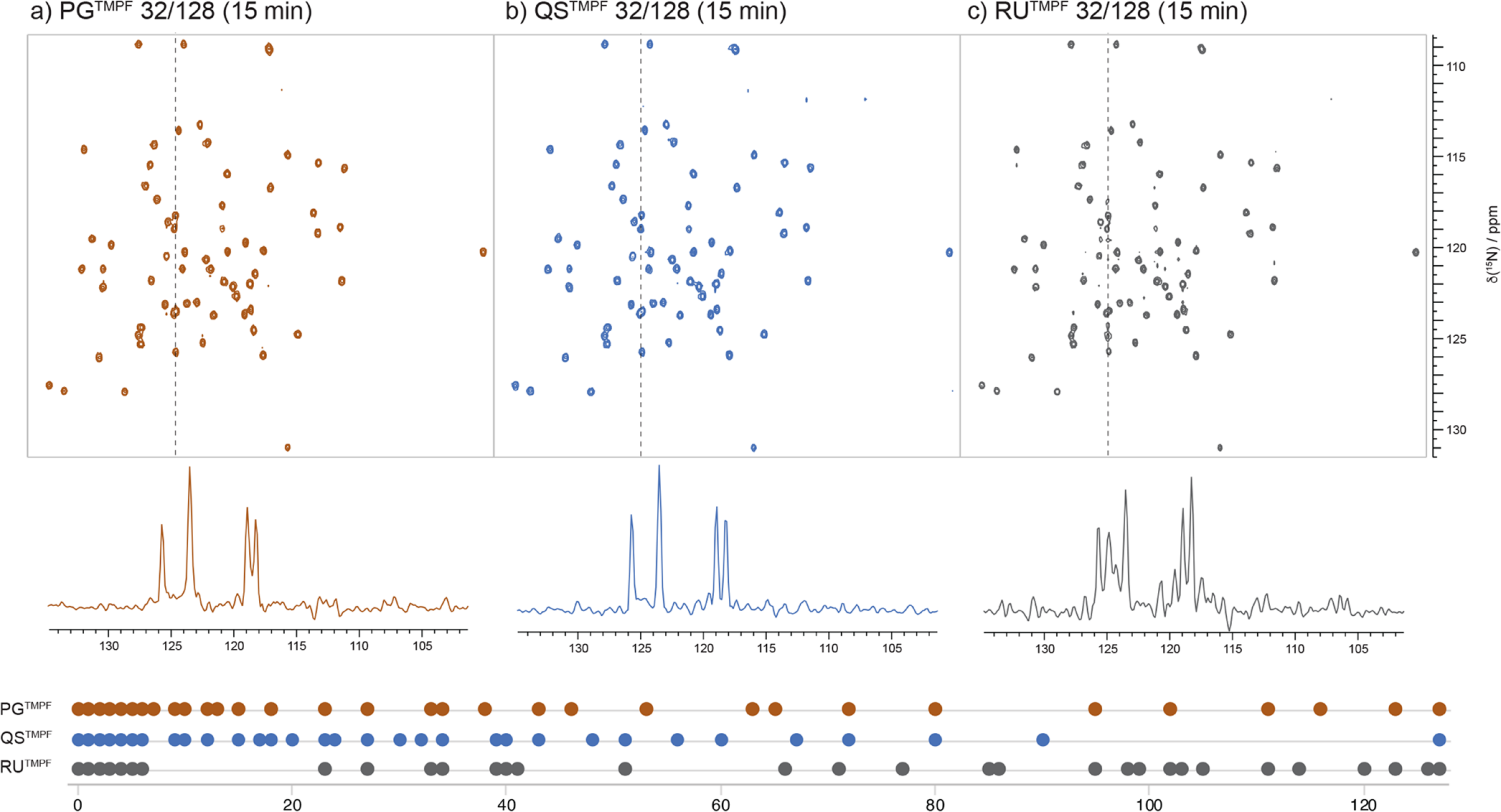
Spectra (NUS/MIST; 32/128; ^1^H­{^15^N}-HSQC; ubiquitin) resulting from the Poisson Gap (PG) and Quantile (QS) algorithms in (a) and (b), respectively, are contrasted with (c) a spectrum employing a random unweighted (RU) schedule (including backfill of 6 samples) that resulted in severe artifactual noise. The dashed vertical lines indicate the positions of the displayed F1 slices, and the schedules are illustrated as well. A large gap occurred early, as well as another nearer the middle, both critical regions, where flaws of this magnitude illustrate the dangers of using RU schedules in sparse conditions, but also show that such large flaws are outside the scope of the TMPF corrective procedure.

Overall, in sparser NUS and when the number of samples was small, weighted sampling (e.g., QS^TMPF^ and PG^TMPF^) was preferred over random unweighted sampling (Figure 7), however Figure 6 reminds that strong RU strategies can still be pursued under other conditions.

## Discussion

The benefits of NUS in multidimensional NMR experiments improve as the sampling becomes sparser, such as for time savings or for distributing samples over longer times for greater resolution. Yet unwanted patterned regions can occur in any schedule (see description in Figure S10, Supporting Information), where in sparser schedules such patterns can represent a significant portion of the schedule and adversely affect subsequent spectral reconstructions. The combined TMPF procedure developed here reduces patterns at local and global levels, and can be applied in automation using default parameters as an “invisible step” in schedule generation.

No instance was observed in which a schedule performed more poorly following the TMPF procedure. The TMPF procedure does not eliminate sampling noise, but is shown to remediate stronger artifacts and helps yield low variance schedules^3^ that perform similarly even with different seeds (e.g., Figure 4). The results of this work (see Methods) are offered in a software package, termed *Usched*, which supersedes the prior *Qsched* program,^6^ and is available now on NMRbox.^33^ *Usched* integrates the TMPF procedure with common scheduling algorithms such as quantiles, Poisson gap, random unweighted, and others.

The TMPF filter led to more pronounced improvements if the initial schedule caused stronger artifacts in reconstructions, but produced minor or indistinguishable effects in some cases. It can be applied to random unweighted (RU) schedules, but initial RU schedules were prone to serious flaws and associated artifacts in sparser regimes that could not always be resolved by the TMPF procedure (e.g., Figure 7). The TMPF method honors user choices while also moving the needle on what conditions can be considered routine, targeting reliable schedule generation on roughly the 20–33% scale, depending on experimental conditions. Recently the RLNE (relative L2 norm error to a uniformly sampled spectrum) and other metrics have shown promise for distinguishing reconstruction algorithms,^45^ but further work is needed to determine if the RLNE has sufficient precision to distinguish among similar schedules of various levels of coherence.

Similar effects of the TMPF method on spectral reconstructions were observed for IST and SMILE reconstructions, supporting the broader utility of rendering schedules decoherent for these widely used methods. However, deep learning (DL)^45–48^ and Hankel matrix methods^49^ use independent algorithms to those considered here, and designing schedules for these new methods may lead to different criteria. Indeed DL shows promise for reducing NUS artifacts,^46,50^ which raised questions such as whether weighted or unweighted schedules may be more beneficial with DL methods.^50^ An in-depth comparison of schedule algorithms and metrics is not available yet for DL methods and is outside the scope of this work, but the new theory and methods here may aid in future efforts such as to interrogate what underlying algorithms are developed in DL models. Note that NUS data may be subjected to time domain analyses.^51,52^

In principle the TM filter operates on local patterns, while the PSF polisher treats global biases (see also Figure S8 of Supporting Information). We did observe the TM filter contributing to global biases in some cases (Figure 1c and Figure S1 in Supporting Information), and we also observed the PSF polisher reverting some of the changes made in the TM filter step such that there is some interaction between these two steps. Overall, the hypothesis that sequential treatments of local and global patterns promotes improved reconstructions is sustained by the data, but future work may be able to further improve these steps and their interplay.

This work supports that the PSF is a useful target for schedule analysis, but requires new approaches to exploit its properties. Our prior work^8^ and this work clarify that the PSF shows poor sensitivity to local patterns. The peak-to-sidelobe ratio remains a fundamental constraint on the schedule,^53^ but is increasingly viewed as a coarse metric, measuring only two points of the PSF, and lacking the sensitivity to characterize schedules.^8,15,27,28^ The approach to PSF smoothing introduced here recasts the PSR as a smoothing metric and treats the PSF (and the underlying schedule) holistically, reducing global biases of schedules where present and yielding improved spectral reconstructions.

Are patterns always harmful in schedules and what should the broader goal of pattern reduction be? This and prior work do not suggest that any attempt be made to reach some arbitrary limit of decoherence, where retaining short patterned regions may even guard against effects of lower harmonics or other flaws.^8^ Better understanding appropriate limits of decoherence, along with improved metrics overall, could stimulate improvements to the TMPF procedure. This work does not aim to produce schedules that would be considered optimal. Variables such as experiment type, hardware specifications, sample stability and concentration, distribution and number of signals, dynamic range, subsequent analyses, usage costs, and more, could all lead to numerous disparate interpretations of the term “optimal” in the context of NUS. Additionally, attempts at optimization based on limited or insensitive metrics risk overfitting a sampling schedule to a particular set of conditions and could lead to unanticipated outcomes if those conditions change.

The use of the TMPF filter can help to make sparser 1D-NUS for 2D-NMR more routine, but several precautions must be mentioned. At lower coverages (i.e., sparser NUS), varying degrees of sampling noise/artifacts are expected to occur regardless of the quality of the schedule and dependent on the greatest number of signals in a given column,^18^ where SNR and the dispersion of the signals likely inform this decision as well.

We showed recently that ultrasparse NUS regimes, on the order of <15% depending on conditions, are improved with schedule decoherence but can still contain substantial noise.^8^ Therefore, the TMPF filter can aid in accessing ultrasparse regimes, but we caution that extreme sparsity remains a risky venture in NUS and often requires bespoke solutions to specific conditions and needs.

A conservative view in this work is to continue the use of short initial uniform regions in weighted sampling,^8^ even though such regions may not be needed when schedules are intrinsically strong. Further work to clarify the utility of initial uniform regions in weighted and unweighted schemes is warranted. Longer uniform regions are also being considered as an approach to challenging dynamic range.^28,54^

We recognized a need to provide a default option for quantile sampling (QS), which was used in all QS cases in this work. Based on this and prior work, the default QS option uses a portion of the sine function as the weighting density, as it balances the advantages of weighting early times with sufficient distribution of samples to preserve line shape such as peak bases^26^ (see also Figure 7). This default “qsin” option is sparser at long evolution times than the default PG scheme, but denser at the middle of the schedule.

A prior schedule-averaging method was devised to balance randomness (decoherence) with adherence to the weighting function.^26^ It converges to quantiles and is included in the new *Usched* software, and may merit further investigation. Finally, the scope of this work does not examine quantification accuracy, such as NUS in 2D-NOE spectroscopy, which has more strenuous criteria and has been examined recently.^18,28^

## Conclusion

It is well-known that 2D-NMR methods are cornerstones of modern chemical inquiry where more demanding 2D-NMR methods have been emerging for many years that benefit from NUS methods.^9^ This work considered how current NUS scheduling methods in sparser regimes can lead to unwanted patterns in the schedule and resulting artifactual spectral noise, which can be reduced by employing the two-stage TMPF decoherence filter developed here. The hands-free TMPF algorithm is grounded in principles of binary sequences (the Thue–Morse sequence and the point spread function) and treats decoherence at both the local and global scale in 1D-NUS schedules to deliver more reliable schedules, and improve the performance of sparser schedules, where predominantly 20% and sample limited schedules were examined. Tests with both IST and SMILE reconstruction support the generality of the TMPF filter.

The fundamental limitation of NUS is that fewer samples represent reduced constraints on the spectral information, and therefore sparser NUS in particular can adversely impact spectral information and artifacts, regardless of the sampling and reconstruction methods.^3^ For example, if too few samples are used for the number of signals expected, then the sampling is fundamentally flawed regardless of the choice of scheduling algorithm. This work does not consider quantification, where tailored NUS workflows should be tested and validated prior to performing *de novo* work. While this work promotes better sampling criteria, opportunities remain for further improvement, from new metrics to potent DL methods.

## Supplementary Material



## Notes

Additional examples and comparisons of TMPF schedule filtering; illustrations of how TMPF filtering changes schedules; principles and algorithms for determining the likelihood of patterns in weighted sampling (PDF)
