# Supplementary material for: Decoherence Principles and Algorithms for One-Dimensional Nonuniform Sampling Schedules for Multidimensional NMR
Source: Anal Chem. 2025 Dec 5;97(49):27091–101. doi: 10.1021/acs.analchem.5c03754 (PMC12713607; doi:10.1021/acs.analchem.5c03754)
Supplement: Supplementary file 1 [file ac5c03754_si_001.pdf]

SUPPORTING INFORMATION for:

Decoherence principles and algorithms for one-dimensional non-uniform sampling schedules for multi-dimensional NMR

Henry B. Rovnyak[2], Lucille E. Cullen[3], David Rovnyak[1,*]

[1] Department of Chemistry, Bucknell University, Lewisburg, PA, 17837
[2] Purdue University, West Lafayette, IN, 47906
[3] Piedmont Virginia Community College, Charlottesville, VA, 22902

*Corresponding: drovnyak@bucknell.edu

Table of Contents **Page**

Table of Contents	Page
Figure S1. Additional Tests of TM and PSF filters with a PG and QS schedules.	S2
Figure S2. Comparisons to a validated schedule and uniform sampling.	S3
Figure S3. Swap distribution for quantile schedules.	S4
Figure S4. Swap distribution for Poisson gap schedules.	S5
Figure S5. Full spectral window of 2D-(^{1}H,^{13}C)-HMBC of strychnine	S6.
Figure S6. Case study of uniform backfilling with random unweighted sampling.	S7
Figure S7. PSF Polisher: Schedule parameter space and stopping condition.	S8-S9
Figure S8. Illustration of treating a patterned subsequence.	S10
Figure S9. Algorithm Design, Efficiency, and Optimization.	S11-S13
Figure S10. Analysis of patterns in weighted sampling.	S14-16
References.	S17

Figure S1. *Additional Tests of TM and PSF filters with a PG and QS schedules.* As described in the main body, the TM decoherence filter acts more locally on the schedule and may tolerate long range residual patterns and, in some cases, could even introduce global biases. The PSF polisher then is applied to treat existing/new global biases.

Whereas this manuscript has employed the 'dynamic IST' option of MNova throughout, the example below (**Figure S1**) employs the default 'static IST' mode of MNova which was more sensitive to aliasing artifacts in this example.

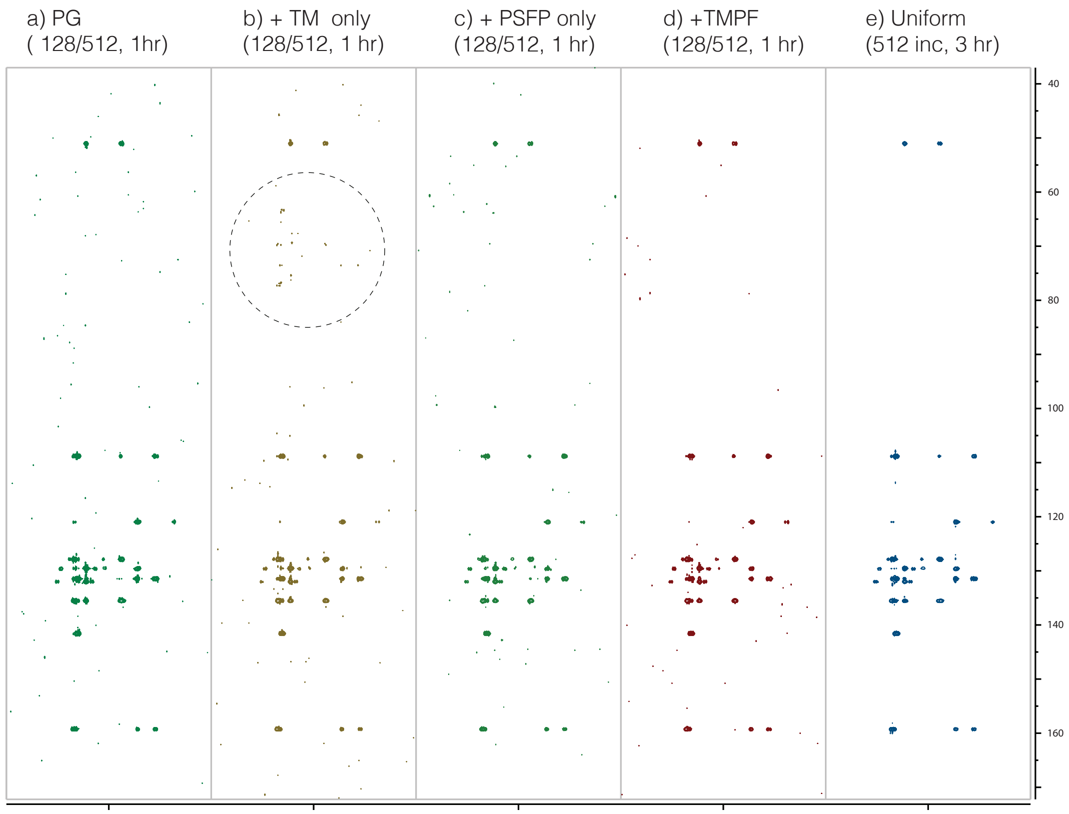

Figure S1. The Thue-Morse sequence and its use as a decoherence filter have the potential to overlook, or even introduce, interrupted (i.e. global, long-range) patterns into the sampling schedule, which are then treated by the subsequent PSF polisher. An example is shown here for a series of ^{1}H-^{13}C HMBC spectra taken on a 9 mM aqueous solution of sodium naproxen. The NUS spectra obtained with default *static* MIST (Mestrenova), whereas all other spectra in this work were obtained with the 'dynamic' IST option. Spectra were normalized for comparison.

A random seed Poisson gap (PG) schedule was generated in *Usched* , modified to include an initial uniform period (16 initial uniform samples) and to guarantee the final sample; it performed well and could be used as-is (**Figure S1a**). Application of the TM filter to that initial PG schedule resulted in weak aliasing artifacts in the spectrum in **(b)**, indicated with a dashed circle, suggesting that some long-range bias had been allowed into the schedule during the TM step. The application of solely the PSFP to the original PG schedule resulted in a spectrum **(c)** that showed no remarkable differences to the original. Finally, applying the full TM+PSFP (aka TMPF) procedure results in the spectrum in **(d)** which corrects the issues that arose in **(b)**. While the original PG schedule performs very well (e.g. no clear sign of aliasing artifacts, low noise/sampling noise), the final schedule in **(d)** may have slightly lower sampling noise.

Figure S2. *Comparisons to a validated schedule and uniform sampling.* Unwanted patterns in sampling schedules that lead to aliasing within the spectral window were the subject of recent work, resulting in the release of fixed schedules that were manually screened with decoherence metrics, had curation of the PSF, validated in 2D correlation experiments, and termed "one-click schedules".[1] We compared the 64/256 "one-click" QS schedule to a new 64/256 QS^{TMPF} schedule, in **Figure S2a**. Results using the new QS^{TMPF} and the prior one-click methods agree well, where the QS^{TMPF} case in **Figure S2a** shows a minor reduction in some of the sampling noise. In practice, NUS is often employed to reach longer evolution times and/or shorten acquisition times, where **Figure S2b** examines a longer maximum evolution increment in a shorter overall time. A 1 hr NUS-QS^{TMPF} HMBC and a 4 hr uniform HMBC, both spanning 512 increments, are overlaid with a horizontal offset for a zoomed signal-dense region in **Figure S2**, comparing well. Both QS^{TMPF} schedules were generated hands-free with default parameters and used as-is, in contrast to the extensive process used to create the one-click schedules.

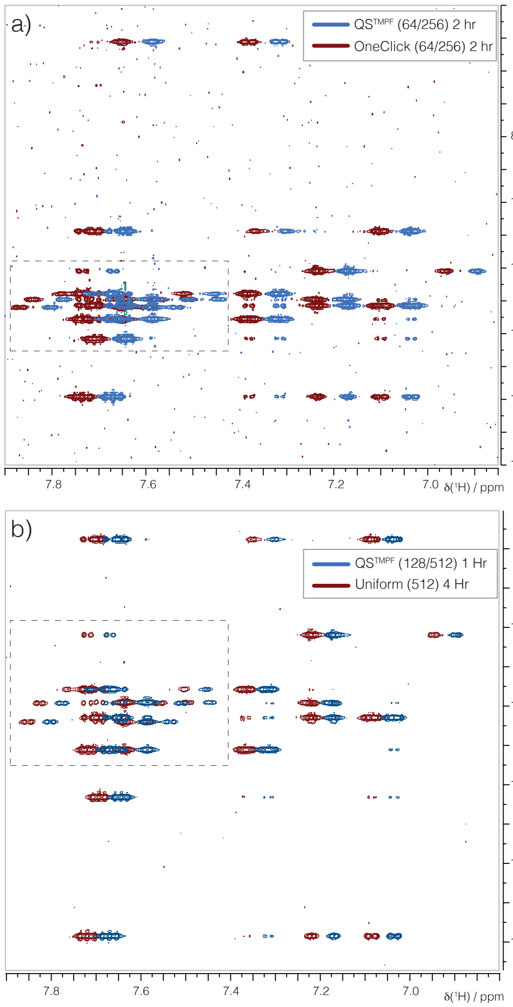

Figure S2. (a) The QS^{TMPF} method was compared to a 'one-click' quantile schedule[1] for NUS $^{1}H(^{13}C)$-HMBC spectra of sodium naproxen (10 mM) for 64x256 NUS, horizontally offset from each other. Squared cosine filters in each dimension and low order polynomial f_2 baseline correction were used (final: 4096x1024). The large f_1 window and lower contours facilitate examination of noise. (b) A 128x512 QS^{TMPF} schedule was used for a 1hr acquisition and compared to a 4 hr uniform acquisition also spanning 512 increments where a similar region to the top panel is depicted. The dashed box highlights the improved f_1 resolution.

Figure S3. *Swap distribution for quantile schedules*.

The distribution of swaps performed by the TM and PSF polisher algorithms is examined for quantile schedules.

The quantile method produces deterministic schedules, so in order to test the two-step algorithm, one fixed schedule was generated and then 10 thousand different randomized initial positions of the TM sequence were used to perform the first TM filter step. Since the initial schedule is deterministic, swaps are localized to the sampling positions, but it is seen that corrective swaps occur early in the schedule and where the sampling is again on the order of 50% of its initial value, regions where patterns in the initial schedules are expected.

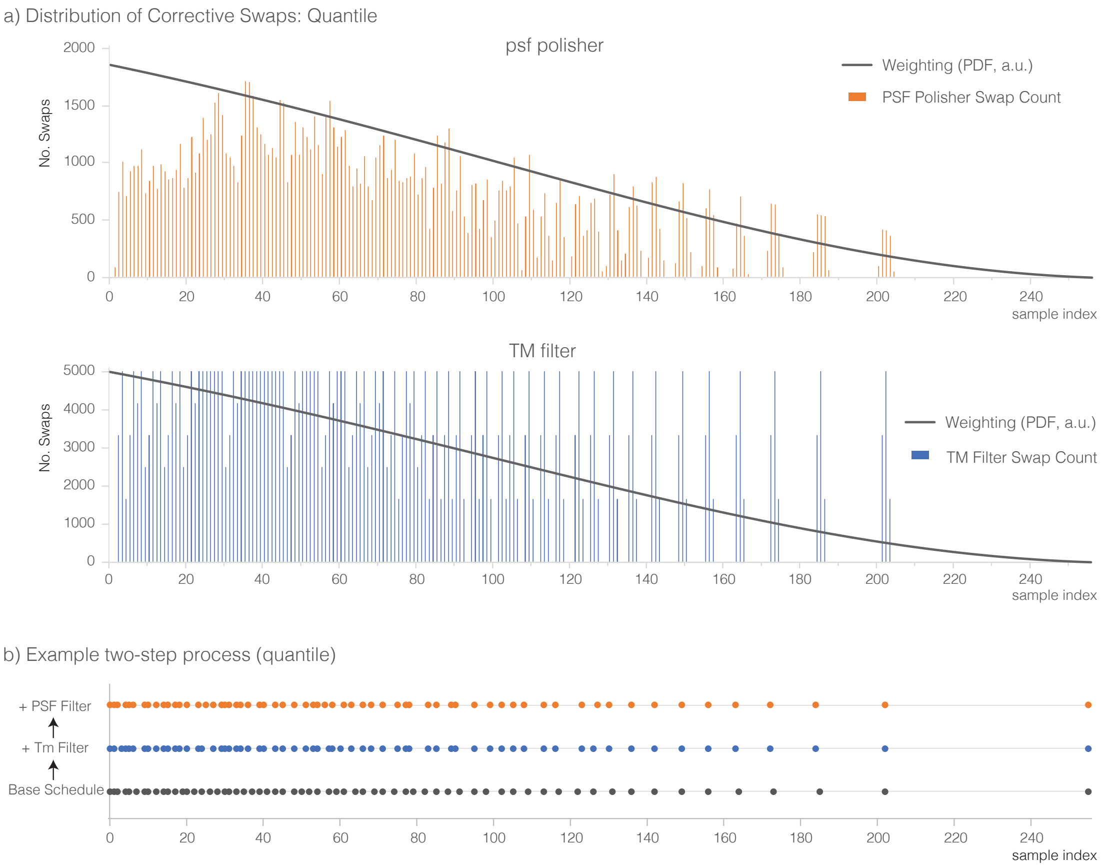

Figure S3. Distribution of corrective swaps by the TM filter and PSF polisher for *one typical quantile schedule that was then treated with 10k different TM filters* (by randomizing the start position in the TM sequence). The discrete appearance of the distribution is due to quantile schedules being deterministic, but the overall trend to apply corrections most frequently early in the schedule is clearly indicated. Since quantile schedules are particularly sparse at the end of the schedule, which is intrinsically decoherent, fewer corrections applied there.

Figure S4. *Swap distribution for Poisson gap schedules*.

The distribution of swaps performed by the TM and PSF polisher algorithms is examined for Poisson gap schedules.

For the PG case, 10 thousand schedules were generated by random seeds and each subjected to the two-step procedure. Since PG uses Poisson variates, samples can occur anywhere in the schedule in principle and therefore corrective swaps may also occur at any position. As in **Figure S3**, notice that swaps are performed most often where the sampling probability is about ~50% of its initial value.

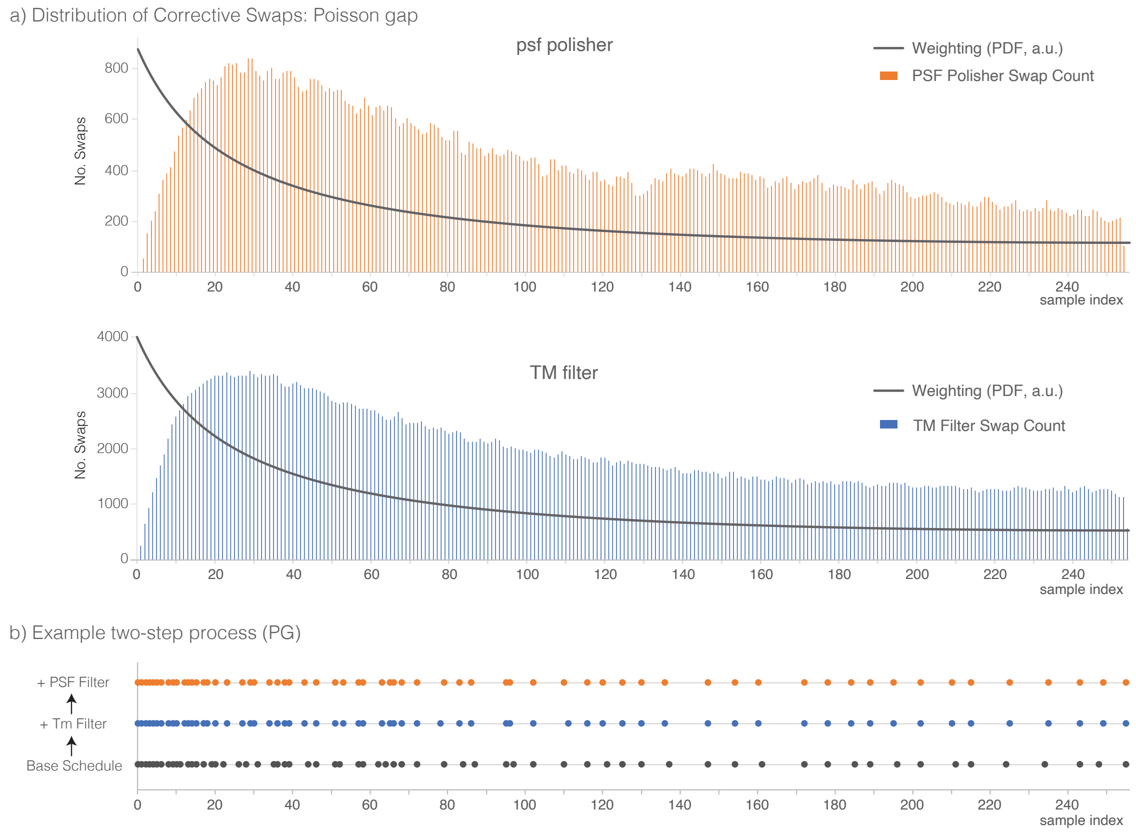

Figure S4. Distribution of corrective swaps by the TM filter and PSF polisher for 10k Poisson gap schedules (according to different random seeds). Corrective swaps are concentrated early in the schedule, particularly when the PDF is roughly half of its starting value. Notice since the PDF remains flat for the majority of the schedule length, that corrective swaps are performed throughout the schedules.

Figure S5. *Full spectral window of 2D-(^1H,^{13}C)-HMBC of strychnine (9 mM, $CDCl_3$, TMS).*

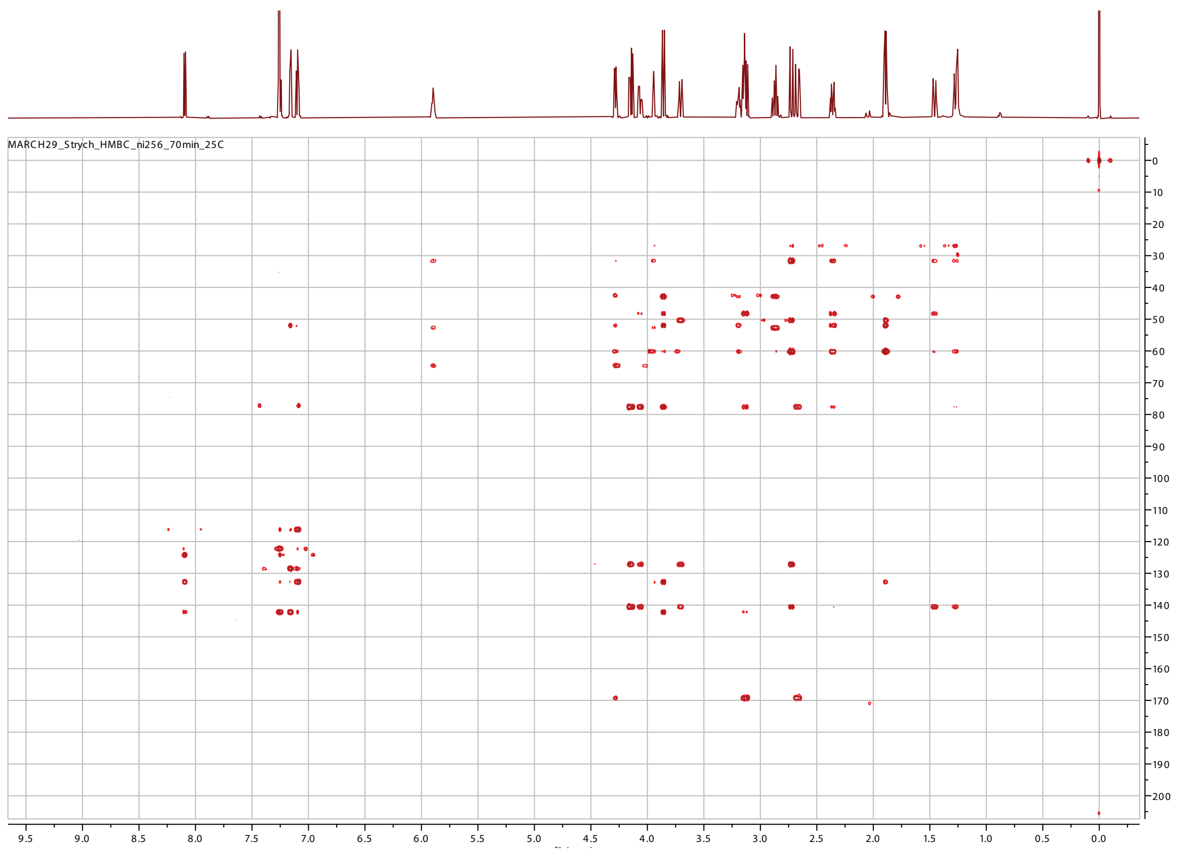

Figure S5. The full uniformly sampled (256 increments) strychnine HMBC is shown to illustrate the different regions excerpted in **Figure 4** of the main text.

Figure S6. *Case study of uniform backfilling with random unweighted sampling.*

Uniform backfilling, along with the application of the TMPF filter, is examined for a case of random unweighted NUS employing 32/128 sparsity. The sparse random unweighted sampling risks large gaps that can occur in early regions of the signal decay.

Some uniform backfill promotes adherence to the Nyquist frequency. Omitting the uniform backfill means that those samples previously tied up in the initial uniform region are then free to be redistributed throughout the schedule, although they are not likely to fall randomly where they are most needed (i.e. in larger gaps).

In the case study below, redistributing the initial uniform points had little effect on the spectra or the background noise. The TMPF method did somewhat improve the background sampling noise and artifacts, but the gaps in the initial schedule were so large that they could not be fully addressed by the TMPF method.

Further work is merited to determine whether uniform backfilling is productive in sparse random unweighted schedules, and how the TMPF method might be further adapted to RU schedules.

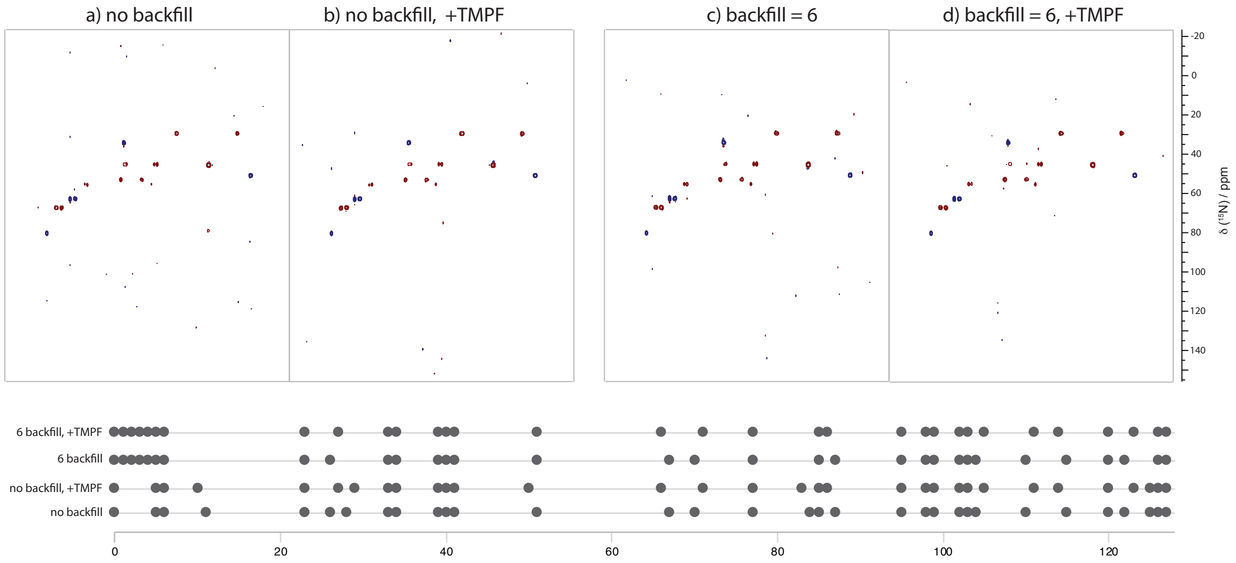

Figure S6. 2D-$^{1}H(^{13}C)$-HSQC spectra of strychnine (9 mM, $CDCl_3$) with the 32/128 schedules illustrated in the lower portion of the figure, processed by MIST.

Figure S7. *PSF Polisher: Schedule parameter space and stopping condition.*

This work did not fine-tune the choices of parameters for the PSF polisher, as the results are insensitive to small changes in these parameters as shown below. Here we show additional tests that measure the effect of the PSF polisher on the PSR for many different schedules with a variety of choices of parameters.

(A)

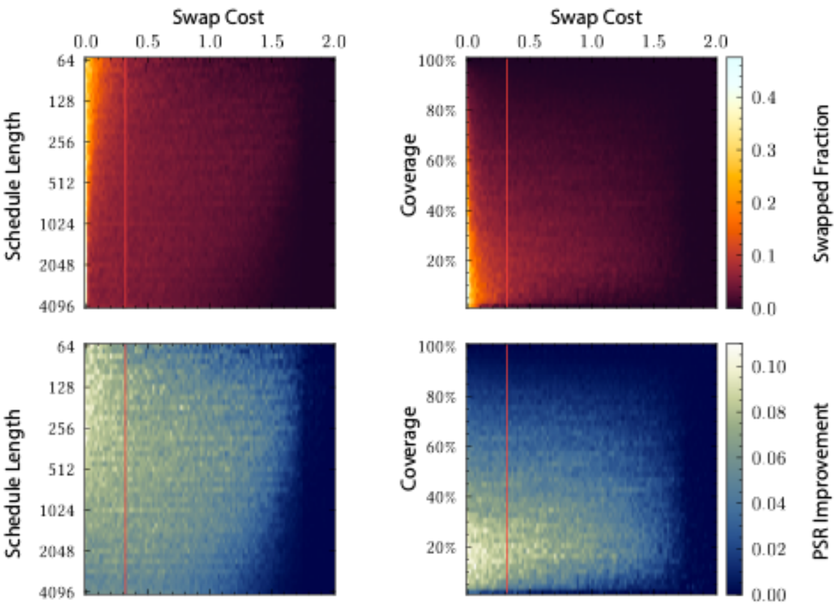

(B)

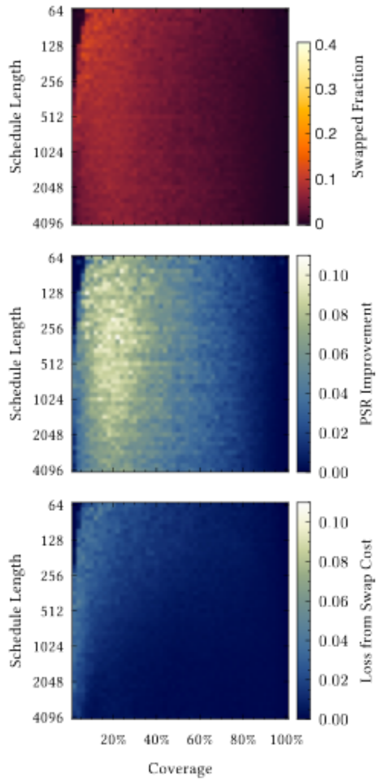

(caption on next page)

Figure S7. (A) The default swap cost parameter (0.32, red line on panels) corresponds to a relatively consistent PSR improvement regardless of the nyquist grid and degree of subsampling (i.e. coverage). (top row) The top row examines the fraction of samples swapped relative to the schedule length and coverage. In the left panel, each pixel is an average of a range of coverages. In the right panel, each pixel is an average of many grid sizes. (bottom row) The bottom row examine the change in the PSR relative to schedule length and coverage. Again, in the left panel each pixel averages many coverages, while in the right panel each pixel averages many grid sizes. Each column is calculated from the same data. **(B)** The effect of the PSF polisher on schedules of different coverages and grid sizes are depicted. In (a) it is generally appreciated that fraction of swapped samples is relatively insensitive to the coverage, but somewhat more changes are made for sparser (e.g. < 40%) schedules. In (b) it can be seen that sparser schedules experience the greatest degree of change in the PSR (again < 40%), where we note again that the polisher both reduces the PSR and is also a smoothing algorithm, where the PSR is taken as a proxy for both of these effects. Finally, in (c) the residual between the final PSR and the global PSR minimum is shown to illustrate that the swap cost parameter still allows the PSFP to approach the global minimum closely and also shows monotonic behavior with no divergences.

In **Figures S7A** and **S7B**, the base schedules were obtained by randomly choosing from Quantiles (with varying PDFs), Poisson Gap, Random Unweighted, and Averaging (exponential with various numbers of schedules averaged), which were selected 38%, 38%, 13%, and 6% probabilities to include more of the commonly used PG and quantile schedules. Next, the Thue-Morse filter was applied using a random subsequence of the TM sequence. Finally the PSF polisher was applied. Each pixel represents an average over 64 schedules generated in this way.

Figure S8. *Illustration of treating a patterned subsequence.*

An excerpt from an actual initial schedule is considered to illustrate the local and global scopes of the TM filter and PSF polisher, respectively.

Notice that despite the extended length of this pattern, it is still a local pattern. Attempting to remediate this pattern with only the PSF polisher is not successful, where the global effect of the PSF polisher is that it only makes one swap in this region. Applying the TM filter first disrupts this pattern, illustrating the specificity of the TM filter to local patterned regions. Finishing with the PSF polisher makes a small number of changes that are based on properties of the entire schedule, not just this region.

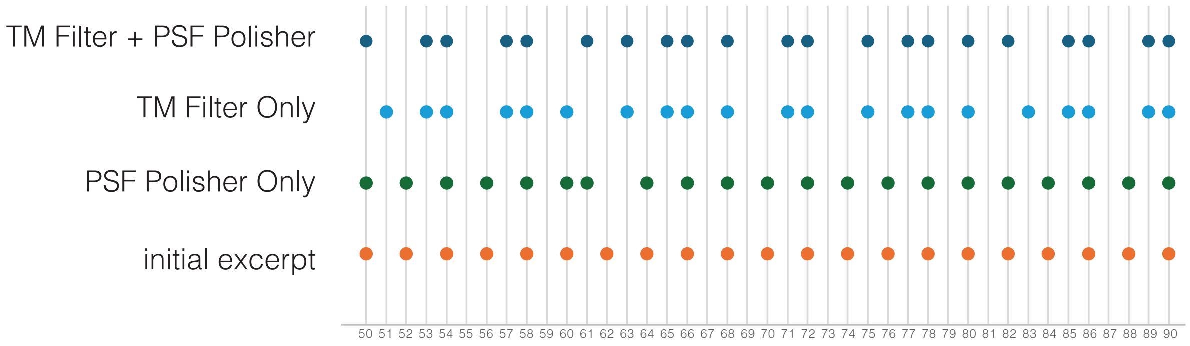

Figure S8. An excerpt of an initial schedule that contained an extremely long 101010… subsequence helps to illustrate the operation of the TM filter and PSF polisher steps.

Figure S9. *Algorithm Design, Efficiency, and Optimization.*

Random weighted sampling: We bring attention to the work of Efraimidis and Spirakis[2,3], who propose an algorithm for calculating a weighted random sample with linear time complexity. First, assign each sample a weight according to the weighting function. Then for each sample, generate a random floating-point number between zero and one and raise it to the power of the reciprocal of the sample's weight. Then, consider the k samples with the largest values to be the weighted random sample. Efraimidis and Spirakis include a proof of correctness.

To select the k largest samples, we can use the Quickselect algorithm by Tony Hoare which partitions a list of values by whether they are greater than, equal to, or less than the kth largest value, whatever it happens to be. It works similarly to Quicksort and operates in linear average-case time complexity. By using these two algorithms together, we can generate a weighted random sampling schedule in linear average-case time complexity.

Thue-Morse filter: Generating the Thue-Morse sequence by iteratively appending the bitwise complement of the sequence to itself is not the most efficient way to do it. There exists a closed form expression of the value of the Thue-Morse sequence for any index i. First, calculate the number of "one" bits in the binary representation of the integer i. This operation exists in most programming environments and is usually called something like "population count", "popcnt", or "count ones". Then, we take that result and determine whether it is even. If the count is even, then the value of the Thue-Morse sequence at index i is 1, otherwise it is 0.

PSF polisher: Removing the central PSF peak is described first. There may be constant offsets or low frequency components of the sampling schedule, such as its weighting function, that would broaden the central PSF peak further. An algorithm should prevent those values from being included in the sorted peak list so that the algorithm does not attempt to flatten the weighting function. A solution to remove the central peak under these constraints (inset of **Figure 2** of main text) is to iteratively remove PSF values in an increasing radius around the central peak, so long as the PSF amplitude of each successive step is monotonically decreasing. The algorithm terminates when it reaches a PSF value with an amplitude greater than the prior one.

After removing the central peak from the PSF, fully sorting the peaks by magnitude is not necessary to determine the threshold. First, because the point spread function is symmetric, we're able to only consider the first half of the elements. Then, we can once again use the Quickselect algorithm by Tony Hoare to determine which element is the nth largest without performing a full sorting operation. Then we can use that as the threshold.

After performing a swap on the sampling schedule, it is not necessary to perform a full FFT to calculate the point-spread function of the new schedule. Instead, we can take advantage of the time-shift theorem and the linearity of the Fourier Transform. Moving a sample point is equivalent to adding a vector of the form [..., 0, 0, -1, 1, 0, 0, ...] to the sampling schedule. The -1 cancels out the existing sample and the 1 moves the sample to the new location. Since the PSF polisher will only ever move a sample by one position, the -1 and 1 elements will always be adjacent. If we want to move a sample earlier in a schedule rather than later, we can negate the vector so that the 1 comes before the -1 instead of after.

This optimization involves precomputing the DFT of the vector [1, -1, 0, 0, ...] where the vector is the length of the sampling schedule. To perform an arbitrary swap, we can apply a linear phase to the DFT of the vector, transforming it into the DFT of the vector that would perform the swap that we would like. Then we can either add or subtract the time-shifted DFT to/from the PSF of the previous sampling schedule to

determine the PSF of the new sampling schedule. We determine whether we add or subtract based on whether we want to move the sample earlier or later in the schedule.

We can also precompute the linear phases because the phase to apply to a particular element will always be of the form $e^(2iπ*k/n)$ where k is an integer and n is the length of the sampling schedule. There are only n possible unique results of this expression, so they can all be precomputed in a list to avoid many trigonometric calculations. These optimizations replace two $O(n\log n)$ operations with $O(n)$ operations in each iteration of the PSF polisher, leaving the computing of the IFT of the thresholded PSF as the only non-linear operation. We do not know tricks to optimize that away, so the PSF polisher is still $O(n^2\log n)$.

PSF Polisher Exit Condition:

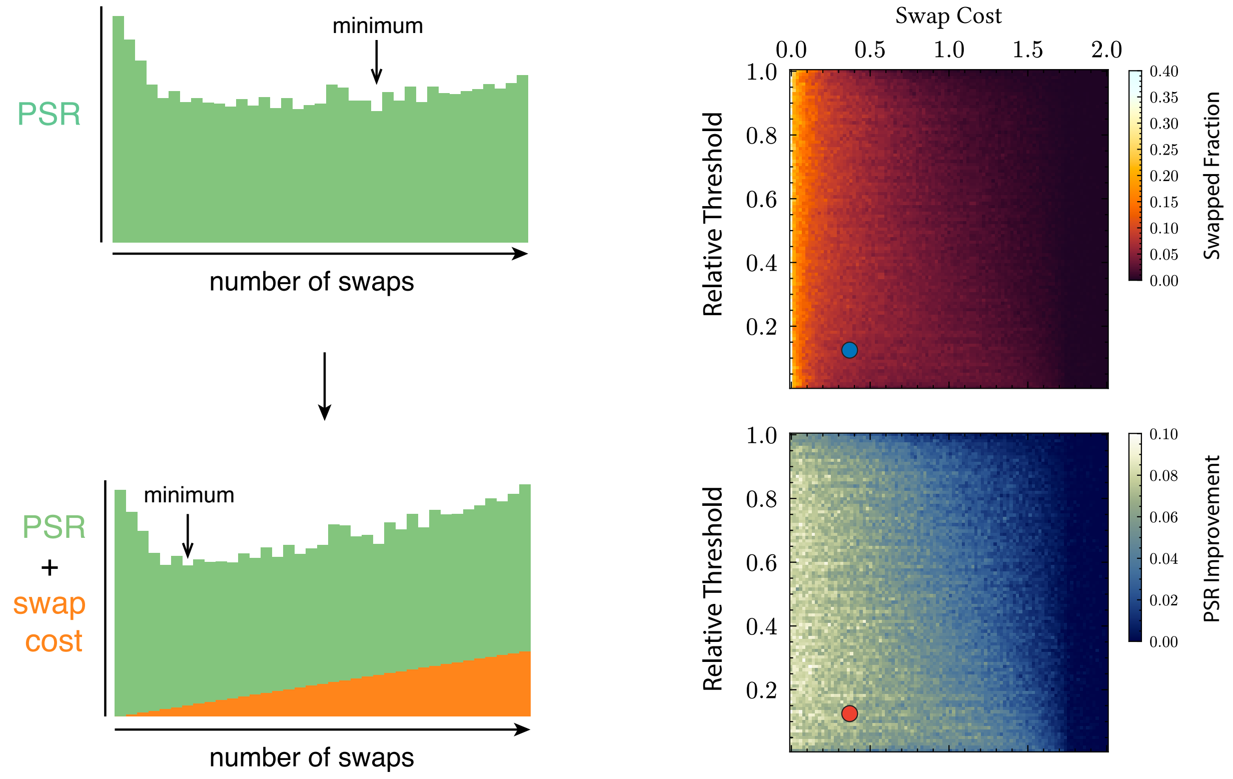

Figure S9. (left column) The role of the swap cost parameter in selecting the number of iterations in the PSFP is illustrated. In the top panel, a global minimum is identified that requires a large number of swaps that could potentially alter the schedule strongly. Combining the PSR with a swap-cost parameter (lower panel) identifies a PSR that is almost as small as the global minimum but is achieved with significantly fewer swaps. (right column) The effect of the PSF polisher on a schedule for a variety of choices of parameters is shown. The top plot shows the number of samples that are swapped as a fraction of the total number of samples in the schedule. The lower plot shows the reduction in PSR after applying the PSF polisher. Each pixel in each plot represents an average over 64 schedules with lengths chosen from {64, 128, 200, 256, 512, 1024, 2048, 4096} and coverages chosen from {50%, 33%, 25%, 17%}, and schedules were randomized over quantile, PG, unweighted, and exponential. The parameter setting used in this paper and set as default in *Usched*, is shown by the filled circles. These panels suggest reasonable defaults (swap cost = 0.32, threshold = 0.1) that are broadly useful, showing that the PSF polisher is effective in improving the PSF with small numbers of changes over a wide range of parameter settings. See Supplemental Information Sections S2 and S5 for additional tests and details.

We further found that the PSF polisher can be applied broadly to diverse scheduling choices (i.e. number of samples and corresponding Nyquist grid) as illustrated for a series of calculations presented in the right column of **Figure S9**, where the default swap cost and relative threshold parameters used in this work and in *Usched* are indicated (see caption for details). It can be appreciated from both panels in **Figure S9** (right column) that the results are relatively insensitive to both parameters and the indicated defaults will be robust to many schedule choices. Still, the results in **Figure S9** (right column) support that users can adjust these values to influence the performance of the PSFP. For example, decreasing the swap cost parameter will lead to more swaps that may result in a slightly lower final PSR.

Since the Fourier transform is power conserving and each sample is assigned an amplitude of 1 prior to computing the PSF, the power of the PSF of a schedule is proportional to the number of samples in the schedule and is constant if the number of samples is not changed. In contrast, it should be recognized that the effect-size of performing a single swap does not change with respect to the number of samples. So as the number of samples increases, the relative effect of an individual swap on the PSR decreases. Therefore, if the swap cost parameter is kept constant, the PSFP would be gentler on larger schedules and harsher on smaller schedules. To address this disparity, it was decided to divide a universal swap cost parameter by the number of samples in the schedule and use that as the swap cost in the filter (i.e. the slope of the orange rectangles in **Figure S7**), which is also convenient for allowing a default value to be reused over many schedule sizes. In other words, the final swap cost parameter used by the PSFP is smaller for larger schedules, allowing for more swaps to be performed in larger schedules in order to reach a minimum. The swap cost can be user specified over the default value.

Figure S10. *Analysis of patterns in weighted sampling.*

The likelihood of patterns occurring in a sampling schedule is considered. In unweighted sampling, patterns can occur anywhere, which can be demonstrated and characterized via the binomial distribution. To treat weighted sampling, a coarse option is to approximate the weighting function (probability distribution function, PDF) as a discrete series of steps and apply the binomial distribution to each constant step. This coarse binomial approximation intuitively demonstrates that certain repeats will occur early in the sampling schedule and near the 50% region of a weighting function (i.e. where repeating (1 0) patterns are most likely). A closer approximation is a discrete calculation of the probability of a target sequence (a kernel density function, KDF) occurring at any schedule location and for any PDF. Both of these approaches to understanding patterns in random weighted sampling are examined here.

When choosing parameters that will ultimately be used to generate a sampling schedule, what is the probability that a specific (and undesired) subsequence occurs in the schedule? This question can be addressed in two cases, where the sampling dimension is taken to be time.

If the schedule is to be *random unweighted*, meaning that samples have the same probability of being selected regardless of where they occur (*i.e.* independent of time), then the probability of a subsequence is the same at every position of the schedule and can be calculated from the binomial distribution.

If the schedule is to be *weighted according to a given probability density function* (PDF), then the probability of encountering a specific subsequence will change over the length of the schedule since the PDF is always changing with time. One option is to model a weighting function as series of steps, each constant over a small range (**Figure S10a**). Applying the binomial distribution to each constant step can estimate the probably of finding a given subsequence in that region, where it can be seen that the alternating pattern (1 0 1 0) is more likely to occur early in the schedule around the region of 50%b probability. As expected, the (1 0 1 0) pattern is less likely as the PDF decreases, and the (1 0 1 0) sequence would also be unlikely where the PDF is very strong (i.e. where 1's have a high likelihood and predominate).

While the coarse model illustrated in **Figure S10a** conveys key principles, a more precise calculation could be performed if we knew the probability of choosing a sample at each position (index *i*) in the schedule, P_i, which is different from its weight. To illustrate, consider an unweighted PDF of length 256, then each sample position has a weight of 1/256; if one is sampling 128 points, then each point has a ½ chance of being selected. We are interested in calculating that chance for any PDF and any number of samples.

If we knew P_i for every position on the Nyquist grid then, by example, we could calculate the probability of the (1 0 1 0) subharmonic sequence at a given location m to be $S_m = P_m(1) \cdot P_{m+1}(0) \cdot P_{m+2}(1) \cdot P_{m+3}(0)$. To generalize, for a kernel $t^{(l)}$ of length l, (e.g. $t^{(l)} = \{1,0,1,0\}$, $l = 4$), the probability of finding the kernel at any position m is

$$S_m = \prod_{k=0}^{l-1} P_{m+k}(t_k^{(l)}) \quad . \qquad (A.1)$$

In order to make use of Eqn (A.1), we must first calculate the probabilities P_i for the given PDF and sampling density. We are unaware of an efficient algorithm to compute these probabilities, so we developed a simple but effective numerical approximation. An algorithm to perform weighted random sampling is to perform weighted random sampling *with replacement*, keeping track of the samples in a list, and then to consider all of the *unique* samples in the list to be our weighted random sample. The algorithm repeats until the expected number of unique samples is found. The approximation does not execute this algorithm, but it

does analyze it. Imagine if instead of repeating the algorithm until selecting the expected number of points, we ran it a specified number of times and kept the samples that are in the list, regardless of how many there are. Then, for any given sample, it would be trivial to compute the probability of it appearing in the list and being selected. It is also trivial to compute the expected value for the number of points selected in total by summing the expected values for each of the points.

The numerical method described here involves performing a binary search on the number of samples to take and searching for a value where the expected number of samples equals the number of samples we would like to take. The closed form solution to determining the probability of a particular sample appearing in the list is defined for fractional values of the length of the list, so the binary search process will allow fractional numbers. This can informally be seen as an expected value for the number of samples with replacement to take before we find the number of samples that we would like. Then, we consider the probability of selecting each sample to be the expected value of finding it in the list where the length is the expected value determined by the binary search.

We find that this method of calculating the probabilities empirically by generating millions of schedules leads to residuals around 1/1000 for very short schedules, and better for longer ones.

Now that we have the computed probabilities of selecting each sample, we can calculate the probability of finding a particular subsequence (a.k.a kernel) at a position m using Eqn (A.1) above, assuming the successive P_i's are independent. By stepping m through every possible Nyquist position, we generate what we call a 'kernel density function' (**Figure S10b**). While this approach is computational, assumes P_i's are independent, and may not be analytically exact, it is superior to the coarse approximation, giving an accurate depiction of where a kernel (i.e. pattern) is likely to appear in a weighted schedule, which would be of interest in schedule design.

One expects the KDF to be low at the beginning of a weighted schedule because there would be few zeros, and low at the end because there would be few ones. Examples of the KDF for different patterns (kernels) are shown in **Figure S10b**.

(**Figure S10** on next page)

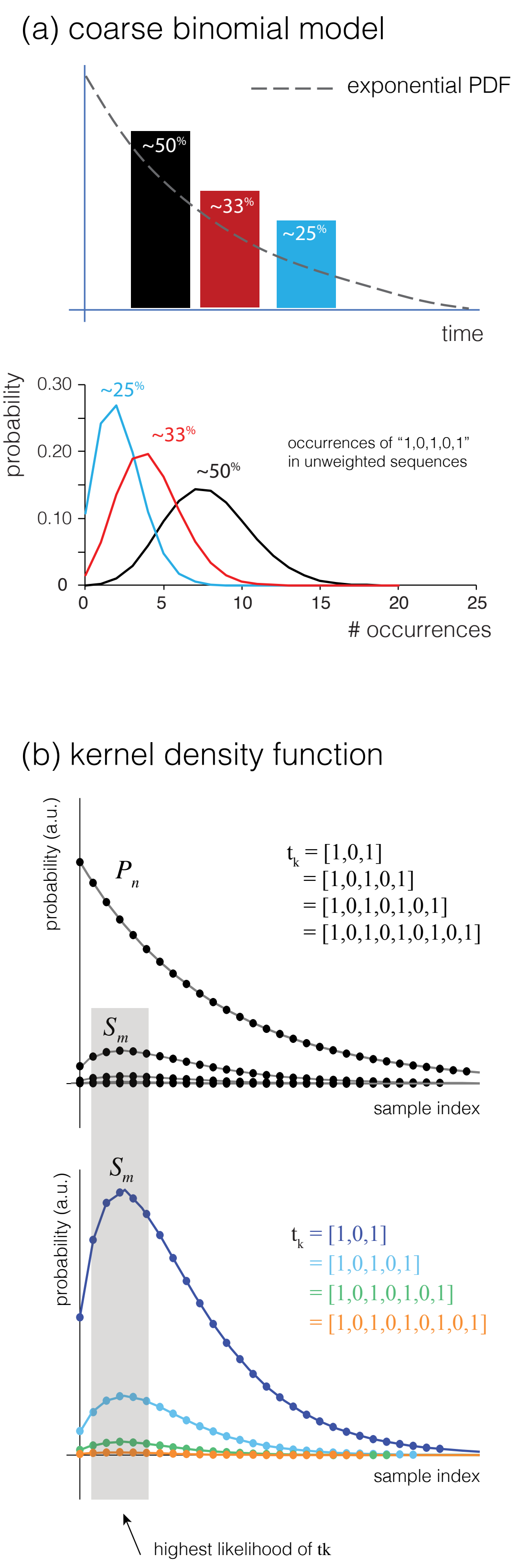

Figure S10: The occurrence of subsequences in random weighted sampling (exponential) is shown for an exponential weighting function and treating the harmful (1 0 1) pattern with different lengths. (a): a coarse model that treats the weighting function as a series of constant steps, allowing the application of the binomial distribution to each step, is illustrated to demonstrate the general concept that harmful repeats such as (1 0 1 0 1) occur more frequently at early times near 50% probability. (b): the kernel density function S_m defined in Eq. (A.1) more precisely defines where the (1 0 1) kernel, also considering different lengths, is most likely to occur. In (b) it is clear that the region of maximum likelihood is very early and has a flat maximum circa 50-60% as would be expected from intuition. In (b) the lower panel is an expansion of the discrete S_m (kernel density) functions of the panel above.

References

(1) Cullen, L. E.; Marchiori, A.; Rovnyak, D. Revisiting Aliasing Noise to Build More Robust Sparsity in Nonuniform Sampling 2D-NMR. *Magn. Reson. Chem.* **2023**, *61* (6), 337–344. https://doi.org/10.1002/mrc.5340.

(2) Efraimidis, P.; Spirakis, P. Weighted Random Sampling. In *Encyclopedia of algorithms*; Springer, 2008; pp 1024–1027.

(3) Efraimidis, P. S.; Spirakis, P. G. Weighted Random Sampling with a Reservoir. *Inf. Process. Lett.* **2006**, *97* (5), 181–185.
